# Characteristics of Intermetatarsal Angle Between the Second and Fifth Metatarsals (M2-M5A) in the Rheumatoid Foot

**DOI:** 10.7759/cureus.24831

**Published:** 2022-05-08

**Authors:** Takaaki Noguchi, Makoto Hirao, Shigeyoshi Tsuji, Yuki Etani, Kosuke Ebina, Hideki Tsuboi, Gensuke Okamura, Shosuke Akita, Seiji Okada, Jun Hashimoto

**Affiliations:** 1 Orthopaedic Surgery, National Hospital Organization, Osaka Minami Medical Center, Kawachinagano, JPN; 2 Orthopaedic Surgery, Nippon Life Hospital, Osaka, JPN; 3 Orthopaedic Surgery, Osaka University, Graduate School of Medicine, Suita, JPN; 4 Musculoskeletal Regenerative Medicine, Osaka University, Graduate School of Medicine, Suita, JPN; 5 Orthopaedic Surgery, Osaka Rosai Hospital, Sakai, JPN

**Keywords:** timed up-and-go (tug) tests, loading axis, intermetatarsal angle between 1st and 2nd metatarsals (m1-m2a), rheumatoid arthritis (ra), intermetatarsal angle between 2nd and 5th metatarsals (m2-m5a)

## Abstract

Background: Increasing of intermetatarsal angle between the first and second metatarsals (M1-M2A) has been reported as a risk factor for recurrence of hallux valgus (HV) deformity, on the other hand, increasing of intermetatarsal angle between the second and fifth metatarsals (M2-M5A) has been reported as a risk factor for resubluxation of the metatarsophalangeal (MTP) joint of the lesser toe after rheumatoid forefoot surgery. In this study, parameters related to increasing M2-M5A were investigated, as compared with M1-M2A and M1-M5A.

Methods: Radiographic parameters including M1-M2A, M1-M5A, and M2-M5A were retrospectively evaluated for 119 lower limbs from 68 patients with rheumatoid arthritis (RA). To clarify the clinical importance of these intermetatarsal angles, relationships with results from the timed up-and-go (TUG) test were also investigated.

Results: M1-M5A showed no correlation with mid-hind foot parameters, whereas M1-M2A and M2-M5A correlated with valgus/varus parameters. An increased M1-M2A was associated with lateral shift of the loading axis in the tibial plafond, whereas an increased M2-M5A was associated with medial shift, but M1-M5A showed no associations. M2-M5A/M1-M2A was significantly lower (1.7) in the normal TUG group than in the delayed TUG group (2.8) (p=0.045).

Conclusions: Different patterns of spread are seen for the forefoot. One has a predominantly increased M1-M2A with lateral shift of the loading point in the tibial plafond, whereas the other has a predominantly increased M2-M5A with medial shift of the loading point in the tibial plafond. M2-M5A also should be calculated, and M2-M5A/M1-M2A might be meaningful in understanding physical mobility in RA patients.

## Introduction

A change in forefoot spread is often seen in hallux valgus (HV) deformity and/or forefoot deformity in rheumatoid arthritis (RA). The intermetatarsal angles between the first and second metatarsals (M1-M2A) and between the first and fifth metatarsals (M1-M5A) have often been measured to evaluate the degree of forefoot spreading. In the previous work of Kushioka et al. [1], it was described that feet with recurrence of HV after surgery showed greater increases in both M1-M2A and M1-M5A preoperatively, whereas increased M2-M5A was a risk factor for resubluxation of the metatarsophalangeal (MTP) joint of the lesser toe after forefoot surgery in the previous work of Etani et al. [2], suggesting that some differences in mechanical conditions may exist between situations of increased M1-M2A and increased M2-M5A. We then hypothesized that predominant spread in M1-M2A might indicate loading on the medial forefoot, resulting in excessive stress on the first MTP joint, whereas predominant spread in M2-M5A might indicate loading of the lateral forefoot, with a subsequent increase in stress on the MTP joint of the lesser toe. In this study, radiographic parameters including the loading axis related to M2-M5A were investigated, as compared with M1-M2A and M1-M5A.

## Materials and methods

Study design and patient population

This observational study retrospectively examined 136 lower extremities from 68 patients with RA. Patients had visited the hospital between December 2016 and January 2019 due to knee, foot, or ankle pain/disorders. Of the 136 lower extremities, 17 were excluded because of a history of surgery on the lower extremity. The remaining 119 extremities were included in this study, and no cases showed ankylosis of any joints in the lower extremities. All patients had been treated with disease-modifying anti-rheumatic drugs (DMARDs) including methotrexate (MTX) and/or biologics to control RA disease activity. The characteristics of patients are shown in Table 1. This research was performed in compliance with the Declaration of Helsinki and has been approved by the IRB of National Hospital Organization, Osaka Minami Medical Center. Informed consent has been obtained from all patients.

**Table 1:** Characteristics of patients Data are presented as mean ± standard deviation (SD). BMI, body mass index; TUG, timed up and go test; TCZ, tocilizumab; IFX, infliximab; GLM, golimumab; ETN, etanercept; ABT, abatacept; CRP, C-reactive protein

	N = 68
Age (years)	67.8 ± 11.5
Male: female (n)	0: 68
Disease duration (years)	21.9 ± 12.9
Weight (kg)	48.9 ± 8.2
BMI (kg/m^2^)	21.4 ± 3.4
Steinbrocker stage (I / II / III / IV) (n)	0 / 9 / 14 / 45
Steinbrocker class (I / II / III / IV) (n)	0 / 42 / 26 / 0
DAS28-CRP score	2.82 ± 0.94
Prednisolone usage (%)	47.1
Prednisolone dosage (mg/day)	1.68 ± 2.29 (0 – 10)
Methotrexate usage (%)	67.6
Biologics usage (%)	27.9
Biologics (n)	TCZ: 6, IFX: 3, GLM: 1, ETN: 4, ABT: 5
TUG average time (seconds)	13.9 ± 11.0

Radiographic assessment

**Figure 1:**
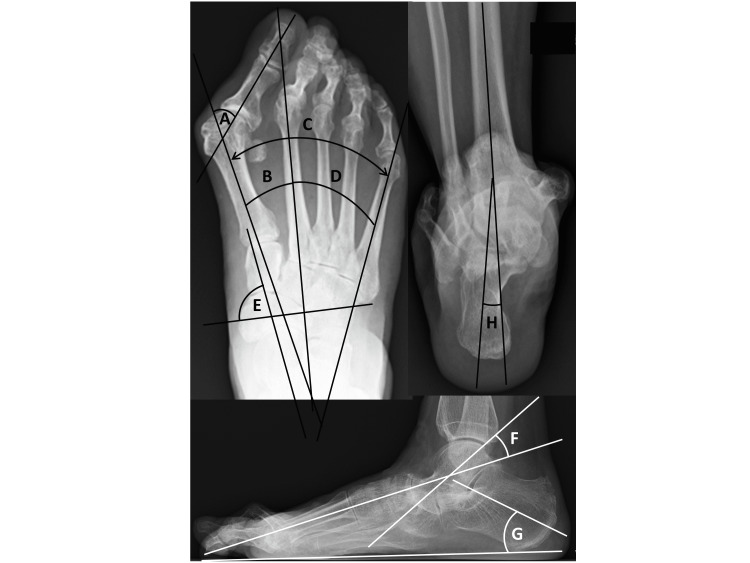
Radiography to measure parameters of foot deformity. Dorsoplantar, anteroposterior weight-bearing radiographs A) Hallux valgus (HV) angle. B) Intermetatarsal angles between first and second metatarsals (M1-M2A). C) Intermetatarsal angles between first and fifth metatarsals (M1-M5A). D) Intermetatarsal angles between second and fifth metatarsals (M2-M5A). E) Pronated foot index (PFI), measured as the angle between the short axis of the navicular and the long axis of the talus (normal, >65°). Lateral weightbearing radiographs. F) Talo-first metatarsal angle (Meary angle). G): Calcaneal pitch angle. Radiographs were taken in a weight-bearing position. Covey method view. H) Tibio-calcaneal angle (TCA).

Dorsoplantar, anteroposterior, and lateral weight-bearing radiographs were taken, and radiographic assessments were performed as described previously [3]. In brief, hallux valgus angle (HVA) and intermetatarsal angles between the first and second metatarsals (M1-M2A), between the first and fifth metatarsals (M1-M5A), and between the second and fifth metatarsals (M2-M5A) were determined as shown in Figure 1. Hardy grade [4] was also measured. Rheumatoid foot usually includes comprehensive foot deformities, so mid-hindfoot parameters were also evaluated. Pronated foot index (PFI) [3] was measured on dorsoplantar weight-bearing foot radiographs as the angle between the short axis of the navicular and the long axis of the talus (normal, ≥65°). The talo-first metatarsal angle (Meary’s angle) [5] and calcaneal pitch were measured on weightbearing lateral foot radiographs to evaluate the level of flatfoot deformity. A radiograph of the subtalar joint (modified Cobey method) [6] was used to measure the tibio-calcaneal angle (TCA), with values ≥2° taken as indicating a valgus state.

**Figure 2:**
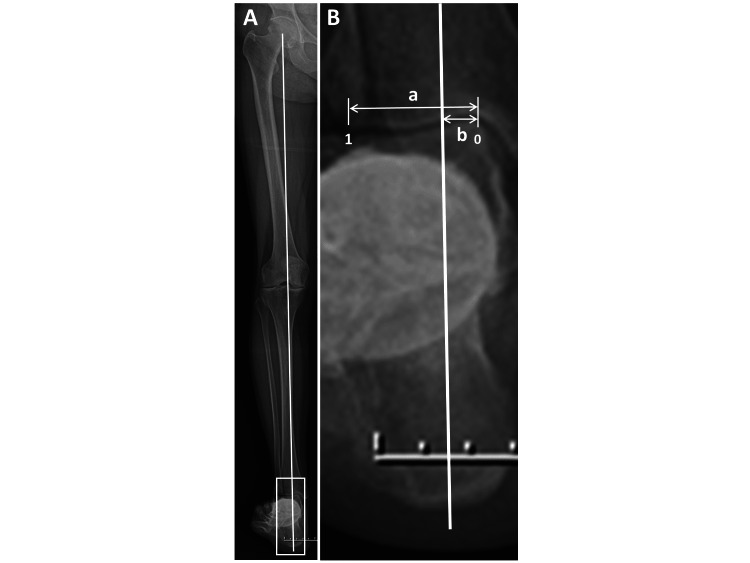
Radiography to measure loading point index (ratio) A) Hip-to-calcaneus radiograph. The solid line shows the loading axis: between center of the femoral head and calcaneal tip. B) Enlarged radiograph of the ankle area (white lined box) shown in panel A. Loading point index is measured as b/a. (From 0 (ratio) [medial end of distal plafond of the tibia] to 1 (ratio) [lateral end of distal plafond of the tibia]).

To check the site of the loading point in the ankle joint, the passing point of the loading axis in the distal plafond of the tibia (loading point index) was measured as b/a in Figure 2, from 0 (medial end of the distal plafond of the tibia) to 1 (lateral end of the distal plafond of the tibia) [3,7], using a radiograph of the hip-to-calcaneus view (HC view), as shown in Figure 2.

Loading transmission to the toes passes through the hip, knee, ankle/hindfoot, midfoot, and forefoot. In this situation, varus knee is associated with development/progression of ankle osteoarthritis [8]. Furthermore, re-alignment of the knee joint has the potential to change the talar tilt angle, subsequently ameliorate ankle pain, and improve foot/ankle function [9], suggesting that knee alignment influences the loading pattern on the ankle joint. Thus, to check knee alignment, hip-knee-ankle (HKA) angle [9] was also measured. A positive HKA angle indicates varus alignment of the knee joint. Values of alignment parameters in this study are shown in Table 2.

**Table 2:** Values for alignment parameters of the foot in this study Data are presented as mean ± SD. Range values are provided in parentheses.

Tibiocalcaneal angle (°)	6.1± 5.4 (-10 – 28)
Pronated foot index (°)	73.3 ± 12.0 (40 – 101)
Talo-1^st^ metatarsal angle (°)	13.4 ± 11.2 (-17 – 51)
Calcaneal pitch angle (°)	15.4 ± 6.3 (-2 – 31)
Intermetatarsal angle between 1^st^ and 2^nd^ metatarsals (°)	12.2 ± 4.9 (2 – 30)
Intermetatarsal angle between 1^st^ and 5^th^ metatarsals (°)	32.4 ± 6.6 (13 – 47)
Intermetatarsal angle between 2^nd^ and 5^th^ metatarsals (°)	20.3 ± 5.6 (7 – 33)
Hallux valgus angle (°)	32.7 ± 19.6 (-4 – 67)
Hardy grade	4.7 ± 2.0 (1 – 7)

Clinical assessment

For clinical assessment, pain was assessed using RA disease activity as evaluated using the DAS28-C-reactive protein (CRP) score [10]. To evaluate physical mobility and static/dynamic balance, the timed up-and-go (TUG) test was performed [11]. Longer TUG time was taken to indicate greater deterioration of physical mobility. Both DAS-28-CRP score and TUG time were investigated at the same time as radiographic analysis in single point or one-shot assessment.

Statistical analysis

Pearson’s rank correlation coefficient was used to investigate the correlations of grades of all combinations of radiographic parameters and clinical assessments in this study using single linear regression analysis. All data are expressed as mean and standard deviation (SD) or median. Differences in measured variables between groups were analyzed with the Wilcoxon signed-rank test or Mann-Whitney U test, as appropriate. These data analyses were performed using IBM SPSS Statistics version 22 software (IBM, Armonk, NY, USA). Values of P <0.05 were considered to indicate statistical significance. Multivariable linear regression analysis with a forward stepwise procedure was performed to analyze correlations. The 95% confidence intervals (CIs) for correlation coefficients were calculated based on Fisher’s z-transformation. A p value <0.05 was considered significant.

## Results

Relationships between loading point index and M1-M2A, M1-M5A, M2-M5A, and other deformity parameters

The loading point index showed significant correlations with M1-M2A (r=0.428), and M2-M5A (r=-0.434), but no correlation with M1-M5A (r=-0.053) (Table 3). Furthermore, the loading point index showed significant correlations with TCA (r=0.446), PFI (r=-0.465), talo-first metatarsal angle (r=0.479), calcaneal pitch angle (r=-0.228), HVA (r=0.26), and Hardy grade (r=0.447). HKA displayed no correlation with loading point index (r=-0.069).

**Table 3:** Correlation coefficients between all combinations HKA: hip-knee-ankle, PFI: pronated foot index, FTA: femoro-tibial angle, TCA: tibiocalcaneal angle, HVA: hallux valgus angle, M1-M2A: intermetatarsal angle between the first and second metatarsal bones, M1-M5A: intermetatarsal angle between the first and fifth metatarsal bones, M2-M5A: intermetatarsal angle between the second and fifth metatarsal bones, DAS: Disease Activity Score

	Disease duration	Age	Loading point index	HKA angle	TCA	PFI	Talo-1^st^ metatarsal angle	Calcaneal pitch angle	HVA	M1-M2A	M1-M5A	M2-M5A	Hardy grade	DAS28-CRP
Disease duration	1													
Age	0.040	1												
Loading point index	0.065	-0.258	1											
HKA angle	-0.073	-0.249	-0.069	1										
TCA	-0.040	-0.254	0.446	0.286	1									
PFI	-0.028	0.204	-0.465	-0.080	-0.368	1								
Talo-1^st^ metatarsal angle	-0.034	-0.073	0.479	-0.200	0.404	-0.290	1							
Calcaneal pitch angle	-0.156	-0.058	-0.228	0.098	-0.202	0.114	-0.594	1						
HVA	0.101	0.082	0.260	0.063	0.330	-0.278	0.105	-0.071	1					
M1-M2A	-0.008	0.108	0.428	0.060	0.369	-0.411	0.250	-0.092	0.666	1				
M1-M5A	0.045	0.205	-0.053	0.168	0.100	-0.170	-0.191	0.147	0.560	0.552	1			
M2-M5A	0.059	0.146	-0.434	0.144	-0.204	0.156	-0.439	0.247	0.072	-0.224	0.689	1		
Hardy grade	0.092	0.108	0.447	-0.059	0.380	-0.458	0.226	-0.105	0.745	0.716	0.331	-0.232	1	
DAS28-CRP	0.058	0.075	-0.189	0.008	-0.251	0.146	-0.158	0.043	-0.015	-0.136	0.204	0.353	-0.209	1

Relationships between M1-M2A, M1-M5A, M2-M5A, and other foot deformity parameters

Both M1-M2A and M2-M5A revealed significant correlations with TCA (r=0.369 and r=-0.204, respectively), while M1-M5A had no correlation (r=0.1) (Tables 4, 5, 6). M1-M2A correlated significantly with PFI (r=-0.411), while M1-M5A and M2-M5A showed no correlations. Both M1-M2A and M2-M5A had significant correlations with talo-first metatarsal angle (r=0.25 and r=-0.439, respectively), while M1-M5A had no correlation (r=-0.191). M2-M5A had a significant correlation with calcaneal pitch (r=0.247), while neither M1-M2A nor M1-M5A showed any correlation. Both M1-M2A and M1-M5A had significant correlations with HVA (r=0.666 and r=0.56, respectively), while M2-M5A had no correlation (r=0.072). M1-M2A, M1-M5A, and M2-M5A had significant correlations with Hardy grade (r=0.716, r=-0.232, and r=0.331). M1-M5A correlated with both M1-M2A (r=0.552) and M2-M5A (r=0.689). Within joint destruction grade, only Larsen grade of the talo-navicular joint displayed any correlation with M2-M5A (r=-0.215).

**Table 4:** Correlation coefficients between M1-M2A and loading point/foot deformity parameters. Data were extracted from Table 3 focusing on intermetatarsal angles and foot deformity parameters. M1-M2A, intermetatarsal angle between first and second metatarsals; M1-M5A, intermetatarsal angle between first and fifth metatarsals; M2-M5A, intermetatarsal angle between second and fifth metatarsals; ADL, activities of daily living; TCA, tibio-calcaneal angle; PFI, pronated foot index; HVA, hallux valgus angle.

	Loading point index	TCA	PFI	Talo-1st metatarsal angle
M1-M2A	r = 0.428 (p < 0.001)	r = 0.369 (p < 0.001)	r = -0.411 (p < 0.001)	r = 0.250 (p = 0.006)
	HVA	M1-M5A	M2-M5A	Hardy grade
M1-M2A	r = 0.666 (p < 0.001)	r = 0.552 (p < 0.001)	r = -0.224 (p = 0.014)	r = 0.716 (p < 0.001)

**Table 5:** Correlation coefficients between M2-M5A and loading point/foot deformity parameters Data were extracted from Table 3 focusing on intermetatarsal angles and foot deformity parameters. M1-M2A, intermetatarsal angle between first and second metatarsals; M1-M5A, intermetatarsal angle between first and fifth metatarsals; M2-M5A, intermetatarsal angle between second and fifth metatarsals; ADL, activities of daily living; TCA, tibio-calcaneal angle; PFI, pronated foot index; HVA, hallux valgus angle.

	Loading point index	TCA	Talo-1st metatarsal angle	Calcaneal pitch angle
M2-M5A	r = -0.434 (p < 0.001)	r = -0.204 (p = 0.026)	r = -0.439 (p < 0.001)	r = 0.247 (p = 0.007)
	M1-M2A	M1-M5A	Hardy grade	
M2-M5A	r = -0.224 (p = 0.014)	r = 0.689 (p < 0.001)	r = -0.232 (p = 0.011)	

**Table 6:** Correlation coefficients between M1-M5A and loading point/foot deformity parameters Data were extracted from Table 3 focusing on intermetatarsal angles and foot deformity parameters. M1-M2A, intermetatarsal angle between first and second metatarsals; M1-M5A, intermetatarsal angle between first and fifth metatarsals; M2-M5A, intermetatarsal angle between second and fifth metatarsals; ADL, activities of daily living; TCA, tibio-calcaneal angle; PFI, pronated foot index; HVA, hallux valgus angle.

	Loading point index	TCA	PFI	Talo-1st metatarsal angle
M1-M5A	r = -0.053 (p = 0.569)	r = 0.100 (p = 0.278)	r = -0.170 (p = 0.064)	r = -0.191 (p = 0.038)
	HVA	M1-M2A	M2-M5A	Hardy grade
M1-M5A	r = 0.560 (p < 0.001)	r = 0.552 (p < 0.001)	r = -0.689 (p < 0.001)	r = 0.331 (p < 0.001)

Relationships between TUG time and M1-M2A, M1-M5A, and M2-M5A

TUG time correlated significantly with M1-M5A (r=-0.326) and M2-M5A (r=-0.401) (Table 7).

**Table 7:** Associations between TUG time and M1-M2A, M2-M5A, M1-M5A, and M2-M5A/M1-M2A (ratio) TUG, timed-up-and-go; M1-M2A, intermetatarsal angle between first and second metatarsals; M1-M5A, intermetatarsal angle between first and fifth metatarsals; M2-M5A, intermetatarsal angle between second and fifth metatarsals.

	M1-M2A	M2-M5A	M1-M5A	M2-M5A/M1-M2A
TUG time	r = 0.032 (p = 0.776)	r = -0.401 (p < 0.001)	r = -0.326 (p = 0.003)	r = -0.098 (p = 0.390)

Evaluation of M2-M5A and M2-M5A/M1-M2A in the analysis using divided time of TUG test

As shown in Table 8, M2-M5A/M1-M2A was significantly lower (1.68±0.64) in the group with TUG test time <11 seconds than in the group with TUG test time ≧11 seconds (2.76±2.30) (Table 5). On the other hand, both M1-M2A and M1-M5A were significantly increased (13.6±4.3 and 34.4±6.6) in the group with TUG test time <11 seconds, as compared with the group with TUG test time ≧11 seconds (10.3±4.9 and 30.3±5.7, respectively).

**Table 8:** Comparison of M1-M2A, M2-M5A, M2-M5A/M1-M2A (ratio), and M1-M5A between TUG time groups divided at 11 seconds. TUG, timed-up-and-go; M1-M2A, intermetatarsal angle between first and second metatarsals; M1-M5A, intermetatarsal angle between first and fifth metatarsals; M2-M5A, intermetatarsal angle between second and fifth metatarsals.

	TUG time ≧11 seconds	TUG time <11 seconds	p
M1-M2A	10.29±4.94	13.61±4.27	0.0011
M2-M5A	20.00±6.65	20.80±4.83	0.76
M1-M5A	30.29±5.68	34.41±6.63	0.0087
M2-M5A/M1-M2A	2.76±2.30	1.68±0.64	0.045

Relationship between M1-M2A, M2-M5A, and M1-M5A

As shown in Table 9 and Table 10, M1-M5A strongly correlated with both M1-M2A andM2-M5A, on the other hand M1-M2A and M2-M5A strongly had a negative correlation with each other.

**Table 9:** Correlation coefficients between M1-M2A and M1-M5A or M2-M5A Multivariable linear regression analysis with a forward stepwise procedure was performed to analyze correlation coefficients M1-M2A, intermetatarsal angle between first and second metatarsals; M1-M5A, intermetatarsal angle between first and fifth metatarsals; M2-M5A, intermetatarsal angle between second and fifth metatarsals.

vs M1-M2A	β	95%CI	p
M1-M5A	1.343	0.993 – 1.007	< 0.001
M2-M5A	-1.149	-1.012 – -0.995	< 0.001

**Table 10:** Correlation coefficients between M2-M5A and M1-M5A or M1-M2A Multivariable linear regression analysis with a forward stepwise procedure was performed to analyze correlation coefficients M1-M2A, intermetatarsal angle between first and second metatarsals; M1-M5A, intermetatarsal angle between first and fifth metatarsals; M2-M5A, intermetatarsal angle between second and fifth metatarsals.

vs M2-M5A	β	95%CI	p
M1-M5A	1.189	0.991 – 1.012	< 0.001
M1-M2A	-0.892	-1.016 – -0.988	< 0.001

## Discussion

**Figure 3:**
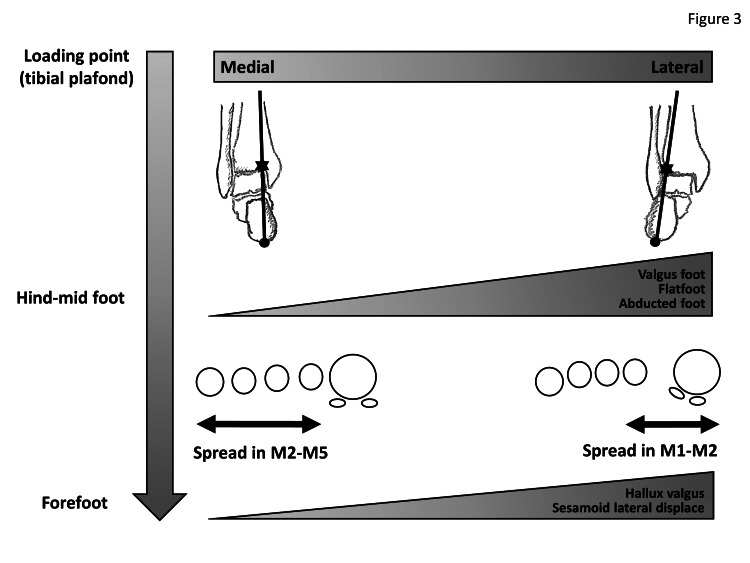
Concept of the scheme through loading axis, mid-hindfoot, and forefoot Figure drawn by author Makoto Hirao

The loading point index showed significant correlations with deformity parameters in the entire foot (TCA, PFI, talo-first metatarsal angle, calcaneal pitch angle, M1-M2A, M2-M5A, HVA, and sesamoid position), suggesting the importance of the relationship between foot deformities and loading point in the tibial plafond. In this situation, interestingly, loading point index showed no correlation with M1-M5A, but correlated positively with M1-M2A and negatively with M2-M5A. At the same time, M1-M5A correlated positively with both M1-M2A and M2-M5A. Furthermore, M1-M2A and M2-M5A correlated negatively with each other. From these observations, the influence of weight-bearing on the spread of M1-M5A is thought to be neutralized by that on M1-M2A and M2-M5A, and two different patterns of spread of forefoot (increased M1-M5A) should be evident: one with a predominantly increased M1-M2A (spread at the medial metatarsals) in the case of lateral shift of the loading point in the tibial plafond; and the other with predominantly increased M2-M5A (spread at the lateral metatarsals) in the case of medial shift of the loading point in the tibial plafond. M1-M5A also showed no correlations with TCA, talo-first metatarsal angle, or calcaneal pitch angle, suggesting that M1-M5A would be poorly affected by mid/hindfoot deformity. On the other hand, M1-M2A correlated positively with TCA, negatively with PFI, and positively with talo-first metatarsal angle. M2-M5A correlated negatively with TCA and talo-first metatarsal angle, and positively with calcaneal pitch angle. At the same time, loading point index correlated positively with TCA, negatively with PFI, positively with talo-first metatarsal angle, and negatively with calcaneal pitch angle. Pes planovalgus (flatfoot) deformity is thus proper to consider categorizing in feet showing lateral shift of the loading axis in the tibial plafond, subsequently contributing to spreading of the M1-M2A and increased HVA and Hardy grade. On the other hand, pes cavus, varus hindfoot, and inversion midfoot deformity are categorized in feet showing medial shift of the loading axis, subsequently contributing to spreading of the M2-M5A. The concepts of these schemes are indicated in Figure 3. From these observations, it is plausible that recurrence of joint subluxation in the MTP of the lesser toe was considered to occur easily in feet with increased M2-M5A [2] and/or varus hindfoot [3]. On the other hand, the associations of valgus hindfoot with HV [12], valgus hindfoot change inducing recurrence of HV [13,14], or correction of valgus hindfoot restoring HV deformity were also considered proper [15].

At present, however, information remains lacking on the clinical features of these intermetatarsal angles. In this study, whether these intermetatarsal angles were associated with physical mobility was further evaluated using the TUG test. RA is one of the diseases causing musculoskeletal ambulation disability symptom complex (MADS) due to structural destruction and inflammatory changes. A TUG test of >11 seconds (elongated) was defined in MADS cases [16]. Although no difference in M2-M5A value was seen between these two groups, M1-M2A was significantly lower (10°) in the MADS group, as compared with the normal group (14°). At the same time, M2-M5A/M1-M2A was significantly increased in the MADS group (2.8), as compared with the normal group (1.7), suggesting that valgus (eversion) foot rather than varus (inversion) foot might have advantages to physical mobility. This may be because loading at toe-off in the stance phase tends to be on the medial toes, including the great toe, as seen in normal gait. Measurement of the M1-M2A or M2-M5A/M1-M2A ratio might offer meaningful information on the physical mobility of patients. Varus (inversion) foot deformity seems disadvantageous for walking, as compared with valgus (eversion) foot deformity. Furthermore, DAS-28-CRP score had positive correlation with M2-M5A (r=0.353) (Table 3), suggesting that joint inflammation should have influences on spreading at lateral metatarsals. Joint luxity in lateral Lisfranc joint might be easily occurred in RA feet. From these perspective, M2-M5A should be discussed in more detail.

As limitations, such investigations should also be performed in non-RA cases, and M2-M5A should be measured in normal feet. Further investigations should be undertaken, including non-spread foot (normal) and spread foot. In any case, an M1-M5A within normal range can be due to decreased M1-M2A with increased M2-M5A, or increased M1-M2A with decreased M2-M5A. Furthermore, abnormally increased M1-M5A might be due to normal M1-M2A with increased M2-M5A, or increased M1-M2A with normal M2-M5A. Transitional change of DAS-28-CRP score and TUG time over time also should be discussed in such studies in the future.

## Conclusions

In conclusion, different patterns of spread foot deformity (increased M1-M5A) were identified: one has a predominantly increased M1-M2A (spread at the medial metatarsals) in the case of lateral shift of the loading point in the tibial plafond, whereas the other has a predominantly increased M2-M5A (spread at the lateral metatarsals) in the case of medial shift of the loading point in the tibial plafond. Furthermore, M1-M5A showed no correlation with valgus/varus foot parameters, while M1-M2A correlated with valgus deformity parameters, and M2-M5A correlated with varus foot deformity parameters. M2-M5A/M1-M2A was significantly lower (1.7) in the normal TUG group, as compared with the delayed TUG group (2.8). Although M1-M5A has been a useful marker for spread foot, the details of this value should be understood by taking into account the loading axis and subsequent mid-hind foot deformity. M1-M2A and/or M2-M5A/M1-M2A ratio might be meaningful for understanding physical mobility in patients with RA.
